# Supplementary material for: Photodynamic Effects with 5-Aminolevulinic Acid on Cytokines and Exosomes in Human Peripheral Blood Mononuclear Cells from Patients with Crohn’s Disease
Source: Int J Mol Sci. 2023 Feb 25;24(5):4554. doi: 10.3390/ijms24054554 (PMC10003466; doi:10.3390/ijms24054554)

Crohn’s Patient #7

Gating Strategy: **(Step 1) Leukocytes (mononuclear cells)**

LED630nm 30 min.

Control

3 mM ALA
4 hr

LED630nm 30 min.

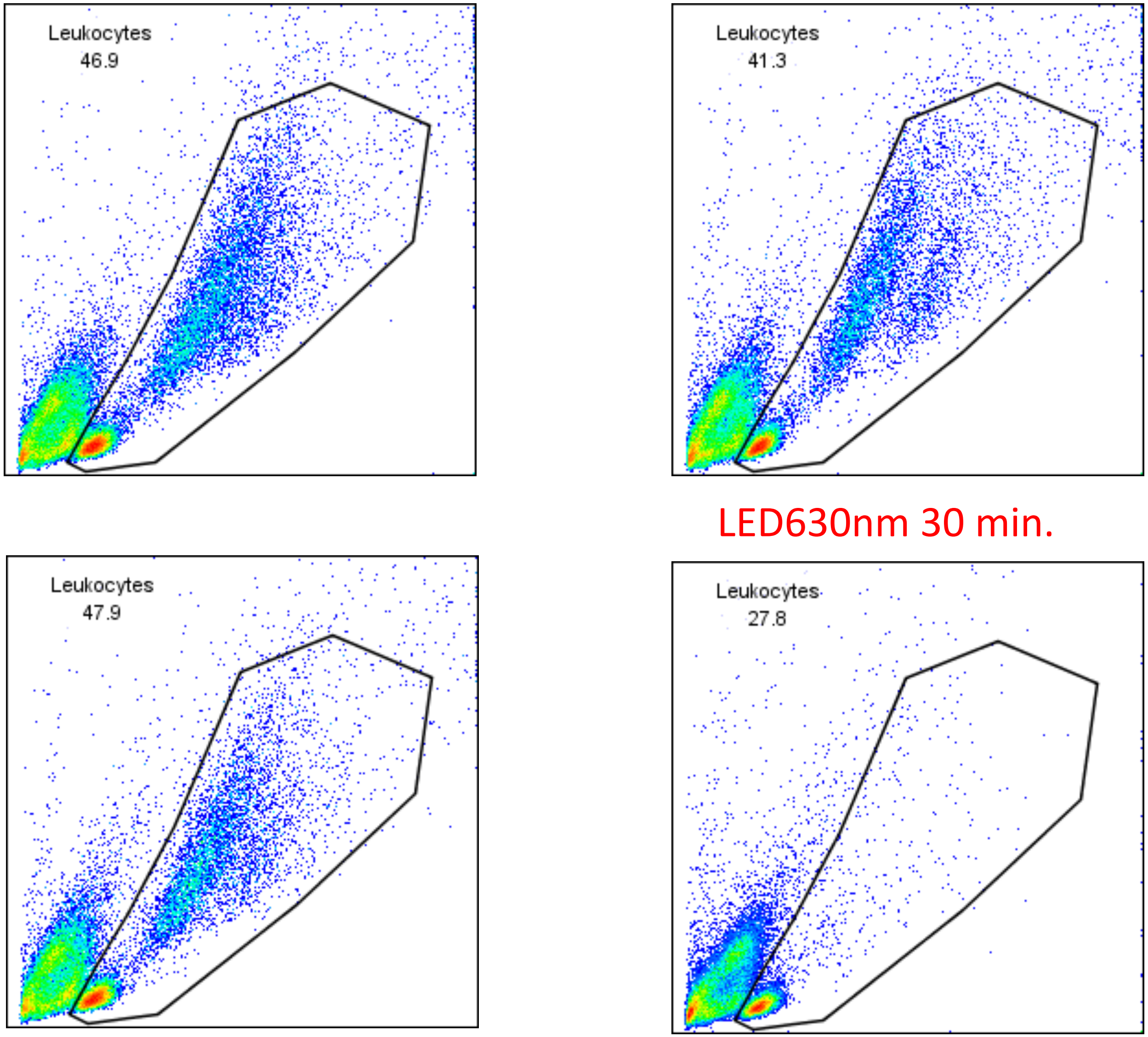

SSC

FSC

Crohn's Patient #7

Gating Strategy: (Step 2) Single leukocytes

LED630nm 30 min.

Control

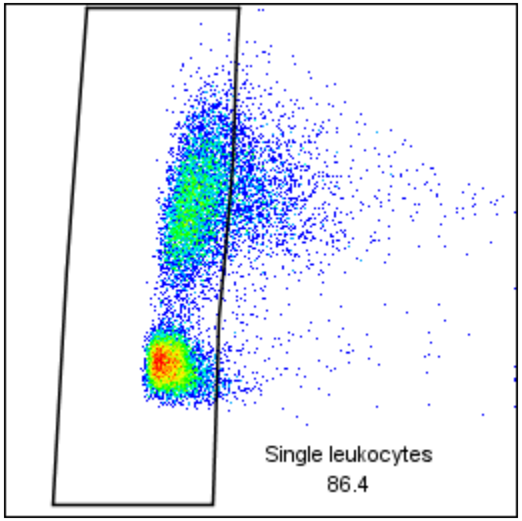

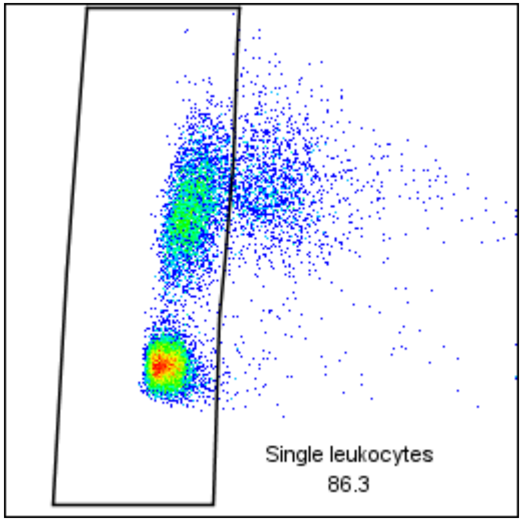

LED630nm 30 min.

3 mM ALA
4 hr

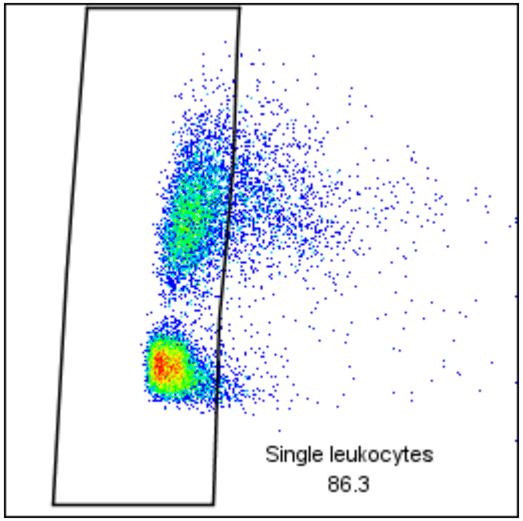

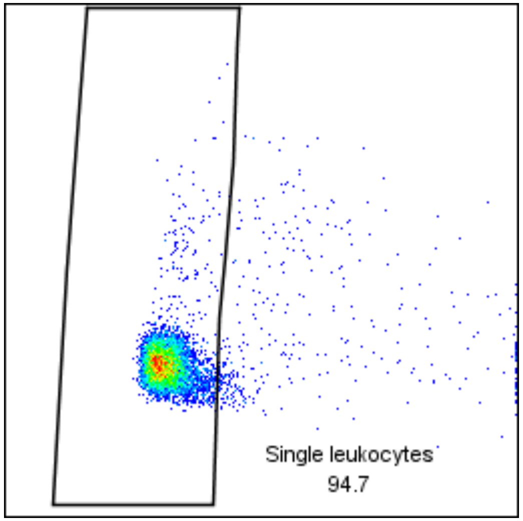

FSC

FSC-Width

Crohn’s Patient #7

Gating Strategy: (Step 3) Lymphocytes and monocytes

Control

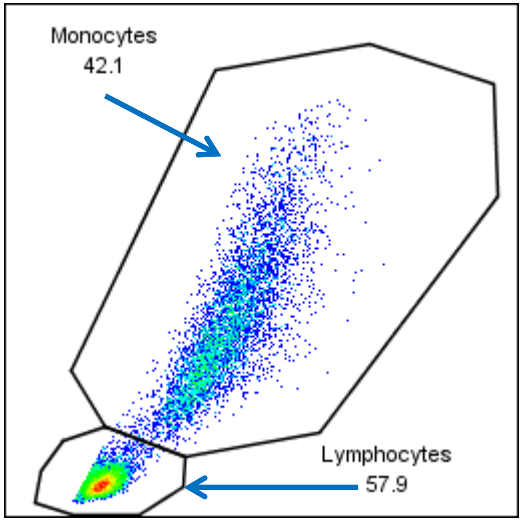

LED630nm 30 min.

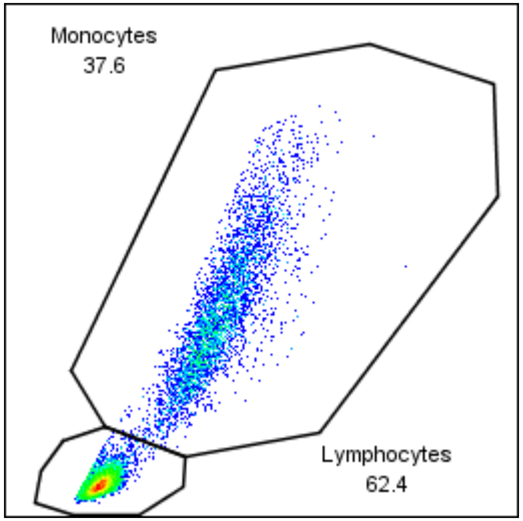

3 mM ALA
4 hr

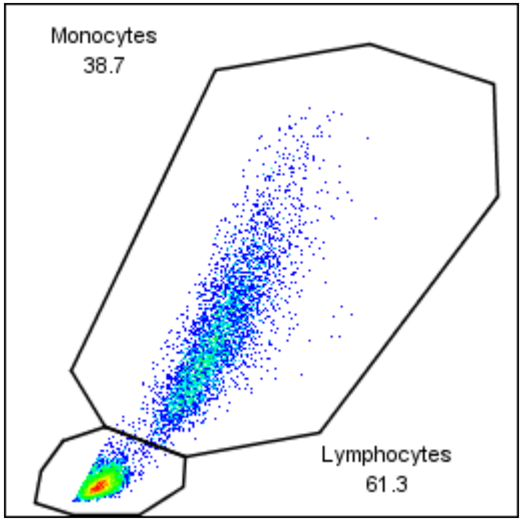

LED630nm 30 min.

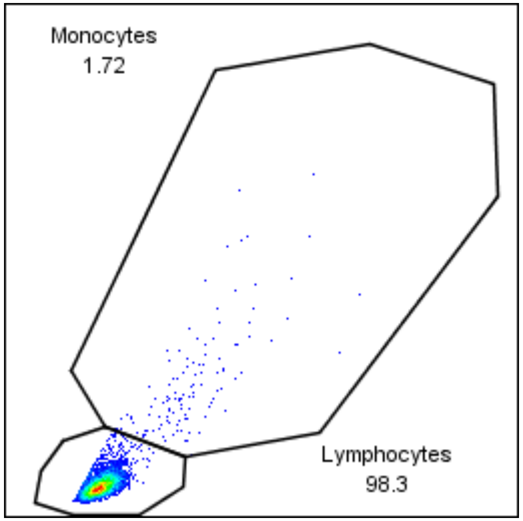

SSC

FSC

Crohn’s Patient #7

Gating Strategy: (Step 4) Live lymphocytes

LED630nm 30 min.

Control

3 mM ALA
4 hr

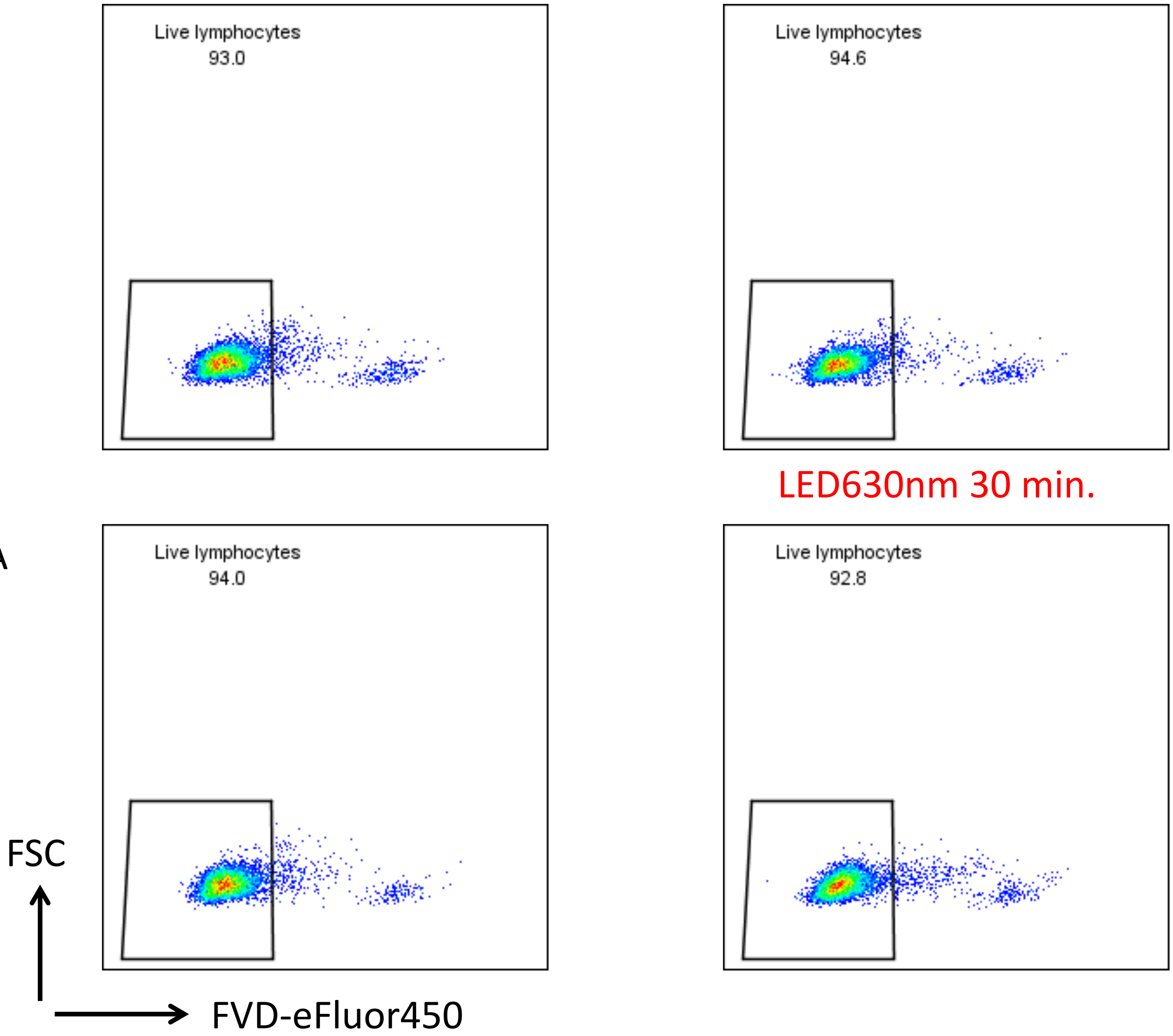

Crohn’s Patient #7

Gating Strategy: (Step 5) Live monocytes

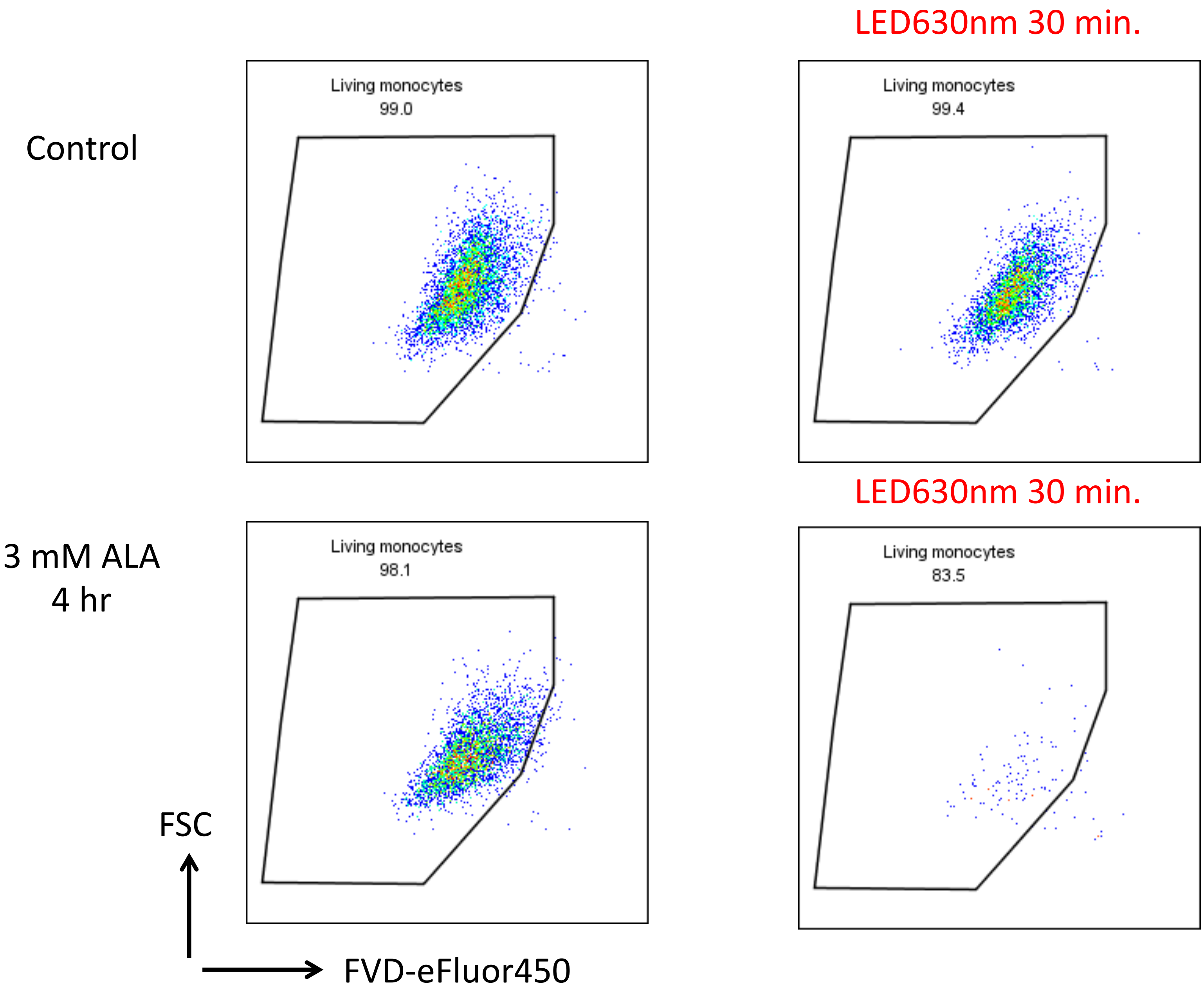

Supplement: Supplementary file 1 [file ijms-24-04554-s001.zip › Figure S1 step 1-5.pdf]
