# Supplementary material for: Photodynamic Effects with 5-Aminolevulinic Acid on Cytokines and Exosomes in Human Peripheral Blood Mononuclear Cells from Patients with Crohn’s Disease
Source: Int J Mol Sci. 2023 Feb 25;24(5):4554. doi: 10.3390/ijms24054554 (PMC10003466; doi:10.3390/ijms24054554)

Crohn's Patient #7

Gating Strategy: **(Step 1) Lymphocytes**

LED630nm 30 min.

Control

3 mM ALA
4 hr

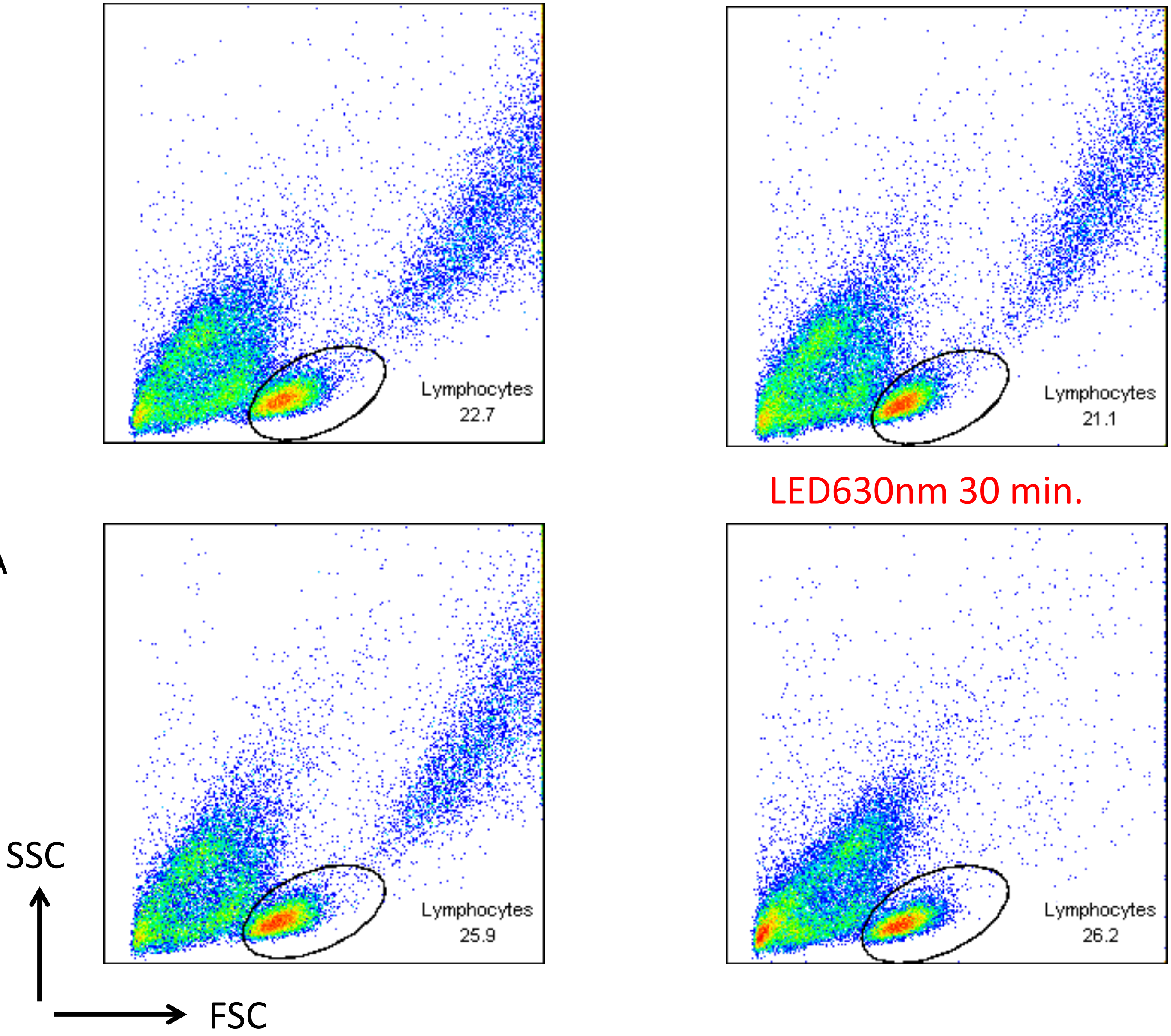

Crohn's Patient #7

Gating Strategy: (Step 2) Single lymphocytes

LED630nm 30 min.

Control

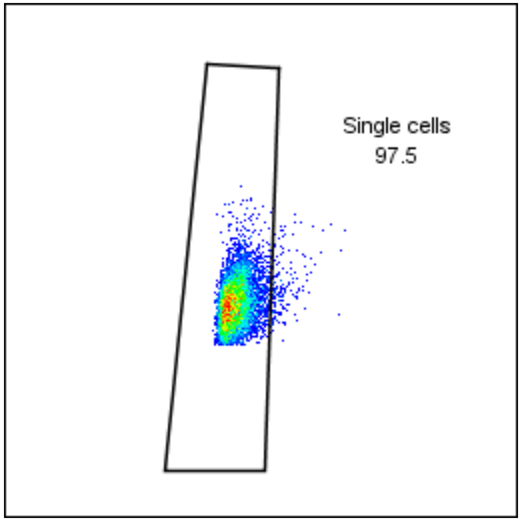

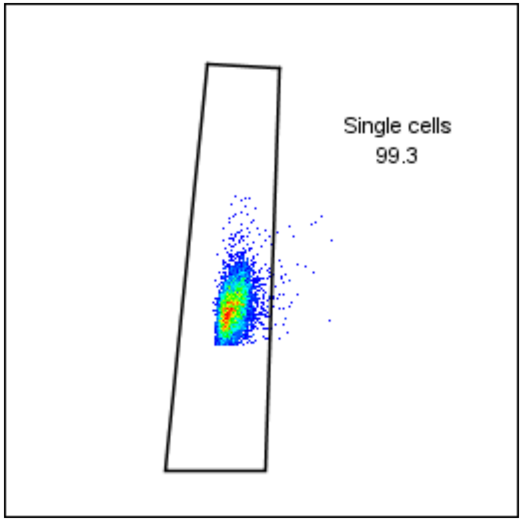

LED630nm 30 min.

3 mM ALA
4 hr

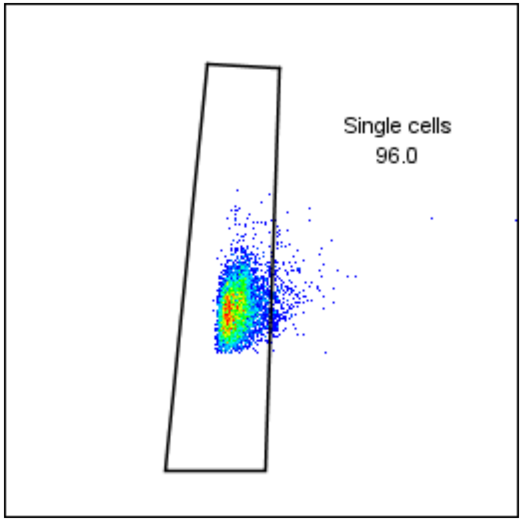

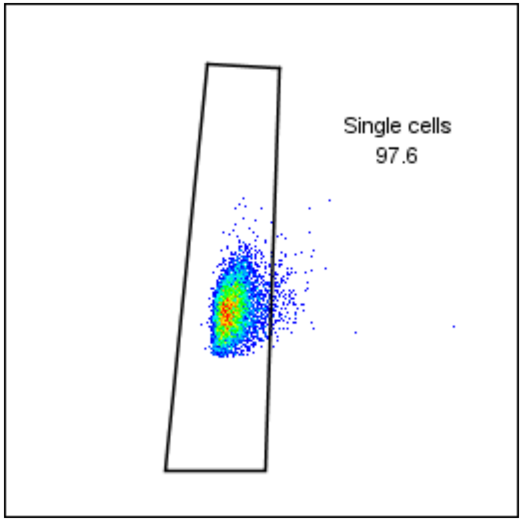

FSC

FSC-Width

Crohn's Patient #7

Gating Strategy: (Step 3) B-cells

Control

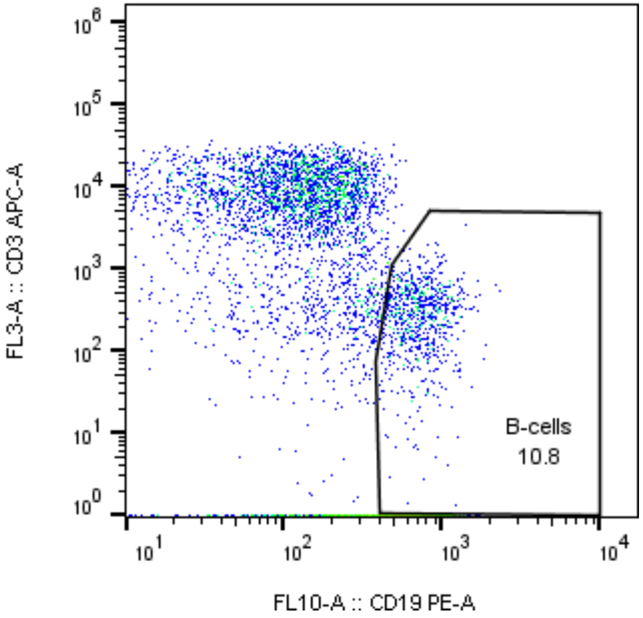

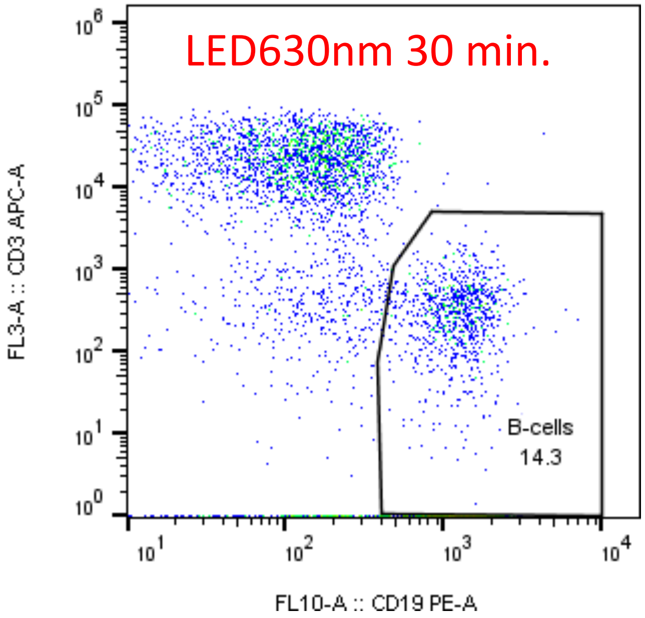

3 mM ALA
4 hr

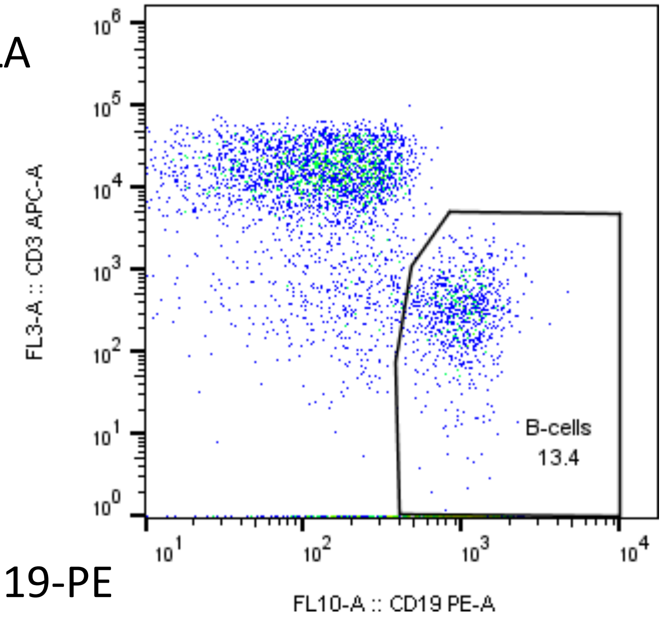

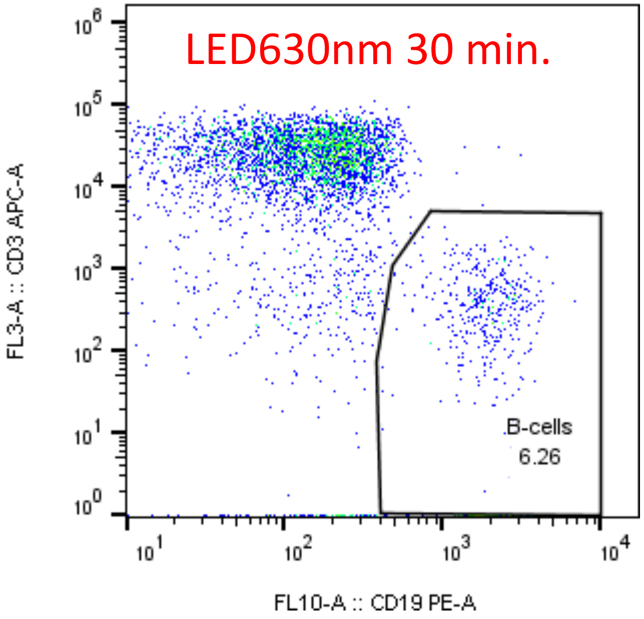

CD3-APC

CD19-PE

Crohn's Patient #7

Gating Strategy: (Step 4) Live B-cells

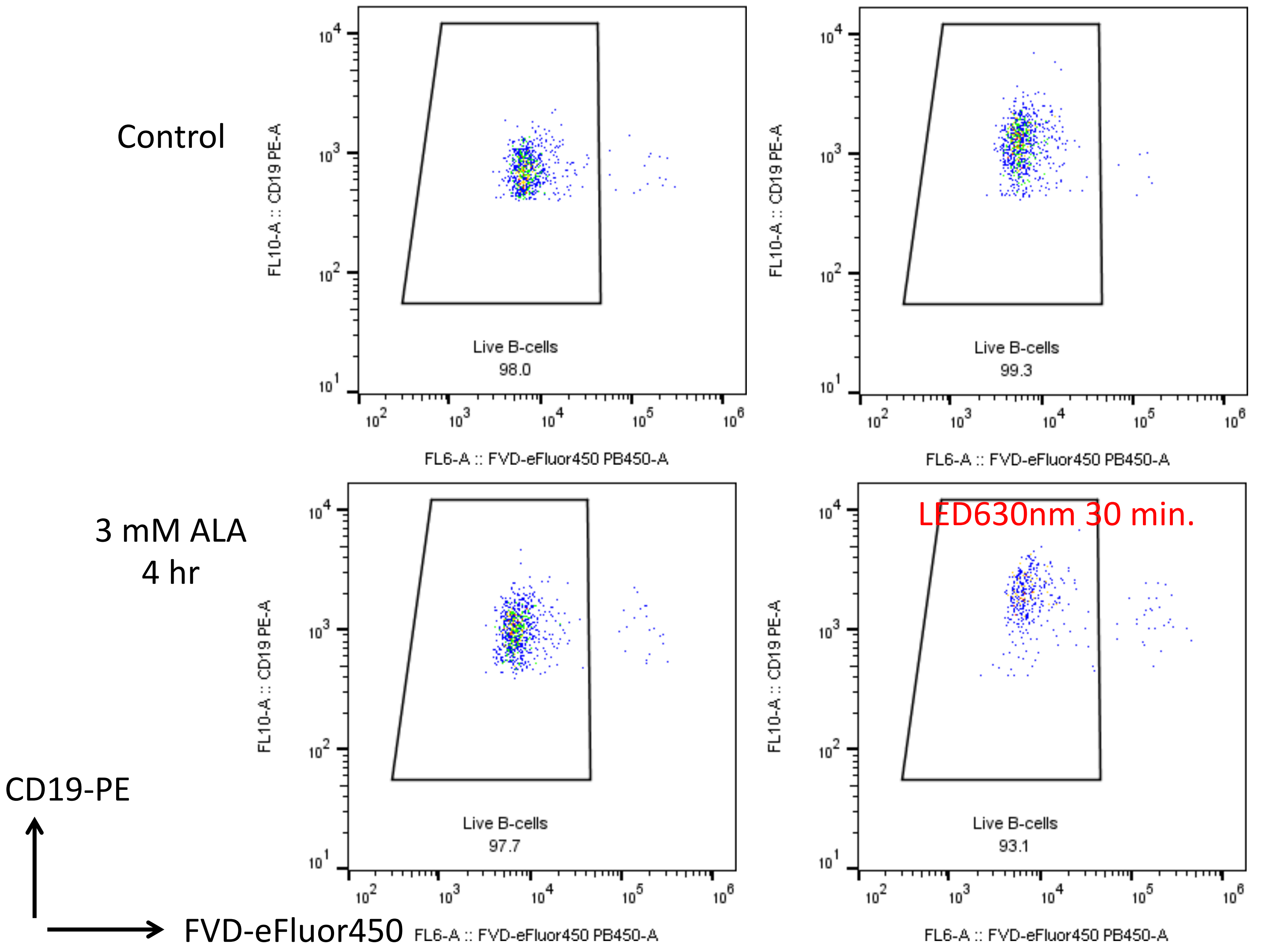

Supplement: Supplementary file 1 [file ijms-24-04554-s001.zip › Figure S2 step 1-4.pdf]
